# Rapid Intervention Through Telephone Encounters for Blood Pressure Control by Residents in an Outpatient Resident Clinic

**DOI:** 10.7759/cureus.3118

**Published:** 2018-08-08

**Authors:** Basil Verghese, Sanjana K Kashinath, Sonam Kiwalkar, Christopher M Henderson

**Affiliations:** 1 Medicine, Rochester General Hospital, Rochester, USA; 2 Internal Medicine, Rochester General Hospital, Rochester, USA; 3 Rheumatology, Oregon State Health University, Portland, USA

**Keywords:** hypertension, resident training, outpatient department, telephone, behavioral medicine, medication

## Abstract

Introduction

Sixty-seven million Americans have hypertensionthat costs the nation $47.5 billion each year. The aim of this study was to determine if regular phone calls by residents helped achieve better blood pressure control.

Methods

The study was a randomized open-labeled study in a resident-run outpatient clinic in Rochester, New York. A total of 57 poorly controlled hypertensives in the clinic were divided into two groups. All the patients received scheduled phone calls once every two weeks for a total of 24 weeks. In one group, the medications were adjusted over the phone and the other group was referred to be seen in the clinic for elevated blood pressures. Both the groups were compared to the usual standard of care group.

Results

Fifty-eight patients were recruited for the trial out of which 53 were used for the final data analysis. Eleven patients completed the trial and had a mean drop of systolic blood pressure (SBP) and diastolic blood pressure (DBP) of 28 and 11 mmHg with p < 0.01 and p < 0.03, respectively. Among the patients who did not complete the trial but answered at least one phone call, the mean drop of SBP and DBP was 29 and 8 mmHg with a p < 0.001 and p < 0.008, respectively. When these were compared to the usual standard of care group, the mean drop in SBP was 28.36 (12.36-48.36), 29.85 (11.85-47.85), and 0.76 (8.04-9.56) with a p < 0.02.

Conclusions

Patients enrolled in the trial had much better blood pressure control compared to the usual standard of care. Residents can take greater ownership of patients to help achieve better blood pressure control. To our knowledge this is the first such study done exclusively by residents in a resident-run clinic.

## Introduction

Seventy-five million Americans have hypertension, which costs the nation $47.5 billion each year [1]. Studies have shown that regular phone calls by healthcare workers or telemetry monitoring can help achieve better patient compliance and blood pressure control [2-10]. Studies have also shown that blood pressures that were more rapidly controlled had a more durable effect compared to others [11]. None of these studies to our knowledge has been done in a resident-run clinic or by residents. Patients in the resident-run clinic are a different population as most of the patients are in the low socio-economic groups and many are without insurance. These patients also have poor compliance and high no-show rates in the clinic making it a challenge to follow up on chronic medical conditions like hypertension and diabetes [12]. The aim of the study was to determine if regular phone calls by residents in a resident-run clinic to patients with poorly controlled hypertension with or without adjustments of oral antihypertensive medications over the phone could help achieve better blood pressure control more rapidly compared to the usual standard of care.

## Materials and methods

The Rapid Intervention through Telephone Encounters for Blood Pressure control (RITE-BP) trial was a prospective, randomized, open-labeled trial that was conducted in the resident-run outpatient medicine clinic at the Rochester General Hospital, Rochester, New York. The study protocol was approved by the institutional review board at our hospital.

Patients were identified and recruited between March 2015 and October 2015. Patients were randomly selected and approached for enrolment when they visited the clinic for a follow up appointment. The inclusion and exclusion criteria were as outlined below.

Inclusion criteria

•       Systolic blood pressure (SBP) of ≥ 150 and/or diastolic blood pressure (DBP) of ≥ 90 as documented in two or more consecutive previous visits to the clinic.

•       Age 25-70 years.

Exclusion criteria

•       If they followed with a cardiologist or a nephrologist

•       If they were followed by the hypertension clinic

•       Non-English speaking patients

•       Did not have a working phone number

•       Documented learning disabilities

•       Pregnancy.

**Figure 1:**
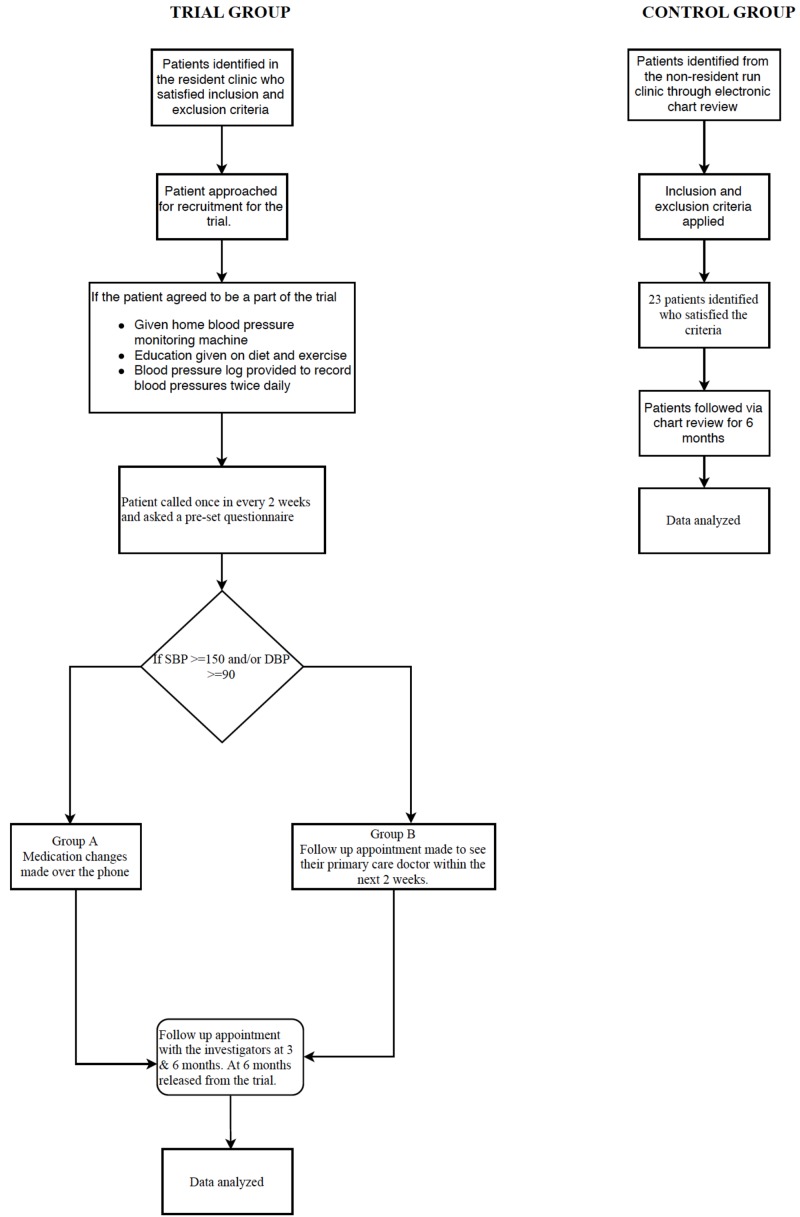
Flowchart of the study protocol.

Once enrolled and after consent was obtained, they were randomly assigned to Group A or Group B. Patients in both the groups received a home blood pressure monitoring kit, a dietary pyramid chart indicating the foods they should be taking to help achieve better blood pressure control, and an exercise chart listing the various aerobic exercises they should try for at least 30 minutes, five times a week. The patients were asked to check their blood pressures twice a day preferably in the morning and evening and record them in a log that was provided to them. For the next six months, patients in both groups received a phone call from one of the investigators every two weeks. During each phone call the patients were asked questions from a pre-set questionnaire. The responses were noted, and the patients’ blood pressure was averaged over a two-week period. If the averaged blood pressure was >150 systolic and or >90 diastolic in Group A, changes were made to their anti-hypertensive medication regimen after discussing with the clinic preceptor. If the averaged blood pressures were elevated in Group B, a follow up appointment was made for them with their primary care provider in the clinic. If a patient did not answer three consecutive calls over a six-week period, they were subsequently excluded from the trial. The patients in both the groups had a follow up appointment with one of the investigators at three months and at completion of the trial at six months at which time their blood pressures were measured, logs documented, and counseling provided again on diet, exercise, and medication compliance (Figure 1).

Patients were also recruited through chart review from the nonresident run outpatient medicine clinic at the same hospital from March 2015 to October 2015 who fulfilled the above inclusion and exclusion criteria and were followed for a six-month period. Patients in this group were labeled as Group C.

Statistical analysis

We used the *t-*test, paired *t-*test, analysis of variance (ANOVA) tests, and Fischer’s exact test for data analysis. Baseline characteristics were compared between three groups of patients with ANOVA tests, Chi-square tests, and Fisher’s exact test. Continuous variables are expressed as mean (M) and standard deviation (SD). Dichotomous variables are expressed as number (n) and percentage (%). The ANOVA analysis was used to compare means for continuous variables. The Chi-square tests were used to compare proportions for categorical variables. The Fisher’s exact test was used for categorical variables when one or more values in the contingency table were below five. To evaluate the efficacy of intervention, paired *t*-test was performed to compare blood pressure values before and after.

## Results

**Figure 2:**
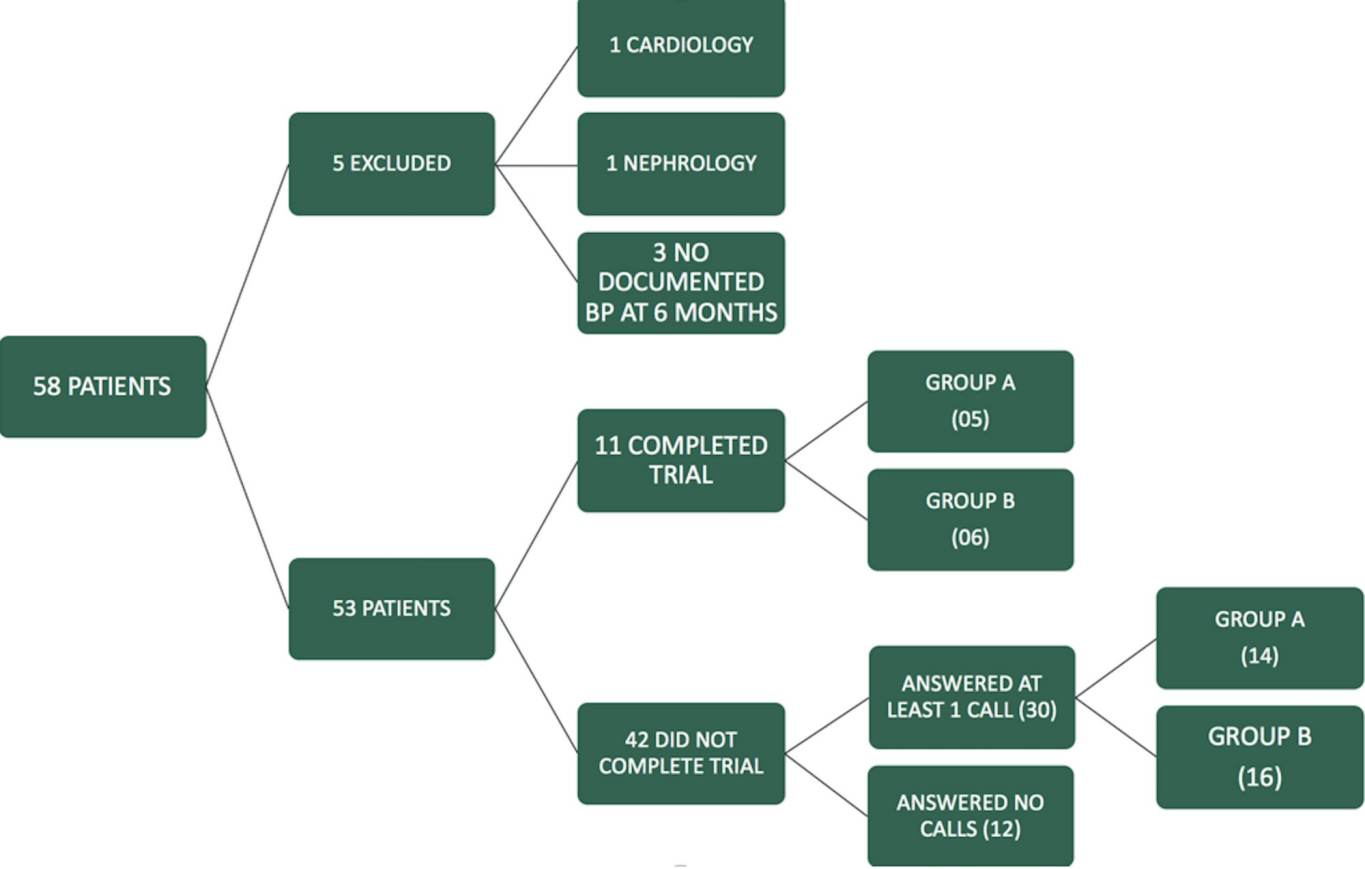
Study groups.

During the recruitment period from March 2015 to October 2015 we approached 183 patients who were eligible for the trial. Of these, 58 patients agreed to be a part of the study. Five patients were excluded from the final analysis, one patient started following with cardiology and another with nephrology for their blood pressure control; three patients did not have a documented blood pressure at the end of six  months (Figure 2).

The baseline characteristics of the study population are outlined in Table 1.

**Table 1:** Baseline characteristics of the various study groups.

Baseline characteristics	Completed trial	Not completed trial	Control Group C	p
	(*n* = 11)	(*n* = 42)	(*n* = 21)	
Mean age in years	59.54	51.32	55	0.64
Sex				
Male %	55	48	47	0.81
Female %	45	52	53	
Race				
African American	2	26	8	0.08
Hispanic	3	8	8	
Caucasian	6	8	5	
Smoking status				
Never	5	14	10	0.07
Former	5	13	2	
Current	1	15	9	
Diabetes mellitus	2	21	3	<0.02
LDL >100 mg/dl	7	10	9	0.65
BMI				
<29	1	11	8	0.82
30-39	9	16	10	
>40	1	15	5	
Systolic blood pressure				
150-159 mmHg	4	14	7	0.89
160-169 mmHg	3	10	8	
>170 mmHg	4	18	6	
Diastolic blood pressure				
< 89 mmHg	6	18	12	0.06
>=90 mmHg	5	24	9	

**Figure 3:**
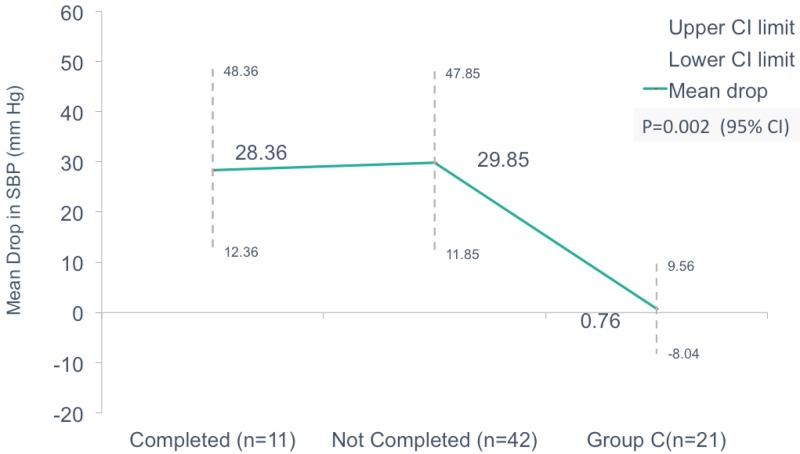
Comparison of mean drop in systolic blood pressure in those who completed the trial, did not complete the trial, and standard of care.

On comparison of the mean drop in SBP (in mmHg) between the groups who completed the trial, those who did not complete and control Group C, we noticed a drop of 28.36 (12.36 to 48.36), 29.85 (11.85 to 47.85), and 0.76 (-8.04 to 9.56) respectively which was statistically significant with p = 0.02 (Figure 3).

**Figure 4:**
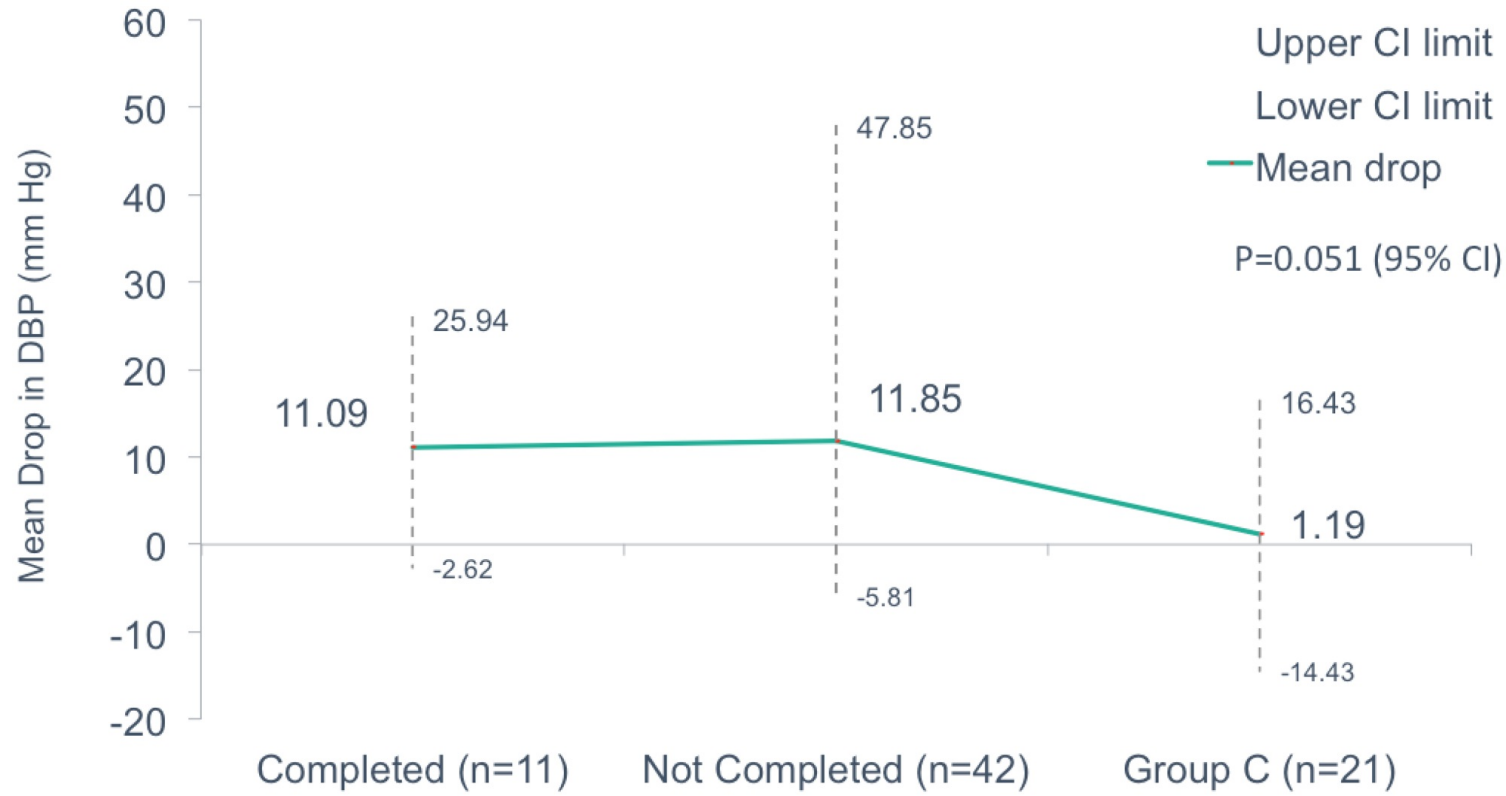
Comparison of mean drop in diastolic blood pressure in those who completed the trial, did not complete the trial, and standard of care.

When we compared the mean drop in DBP (in mmHg) of the same groups we found a mean drop of 11.09 (-2.62 to 25.94), 11.85 (-5.81 to 47.85), and 1.19 (-14.34 to 16.43) for those who completed the trial, not completed the trial, and the control Group C, respectively, with a p = 0.051 (Figure 4).

**Figure 5:**
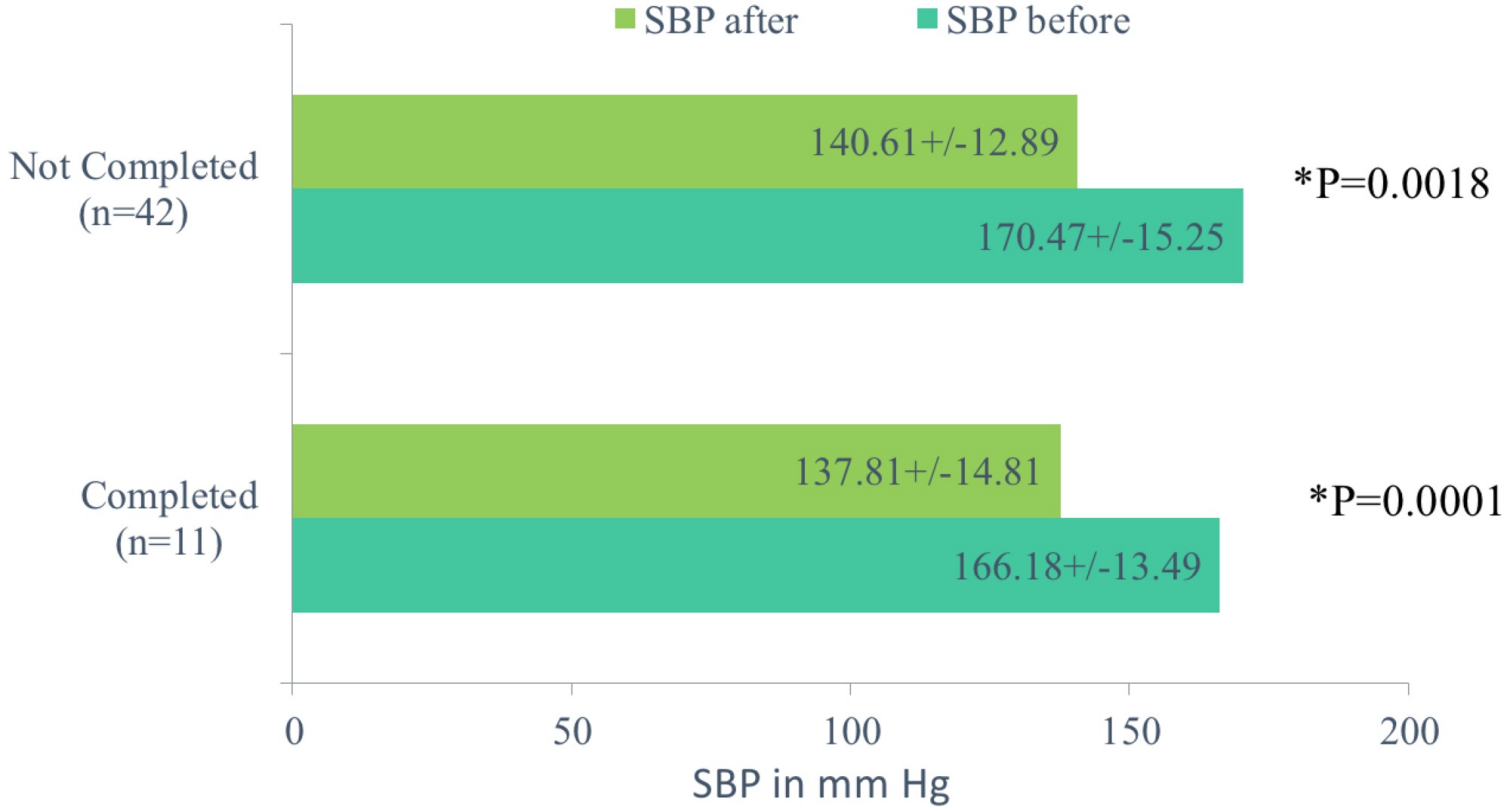
Comparison of mean systolic blood pressure in those who completed the trial and those who did not complete the trial.

**Figure 6:**
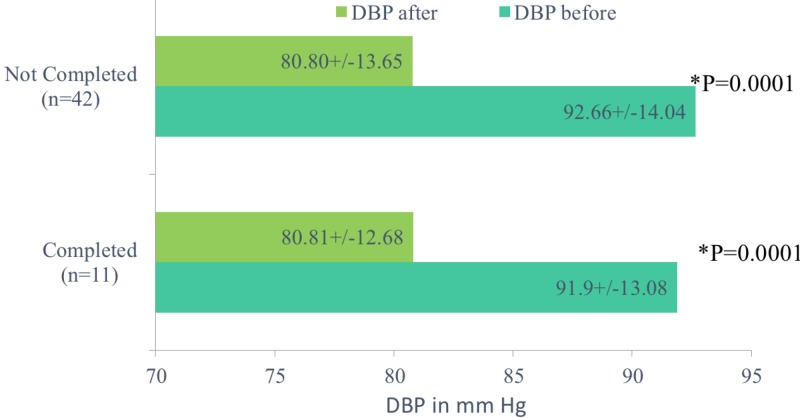
Comparison of mean diastolic blood pressure in those who completed the trial and those who did not complete the trial.

We then proceeded to analyze the drop in SBP and DBP in those who did not complete the trial and those who did complete the trial. In those who did not complete the trial, the mean SBP (in mmHg) before the trial and at the completion of the trial was 170.47 +/- 15.25 and 140.61 +/-12.89 with p = 0.018 and the mean DBP was 92.66 +/- 14.04 and 80.8 +/- 13.65 with p = 0.001, respectively. For those who completed the trial, the mean SBP (in mmHg) before and at the completion of trial was 166.18 +/- 13.49 and 137.81 +/- 14.81 with p = 0.0001 and mean DBP was 91.9 +/-13.08 and 80.81+/-12.68 with p = 0.0001, respectively (Figures 5-6).

## Discussion

One particular area that continues to be neglected is the resident-run clinics when it comes to blood pressure control. These resident clinic patients are usually from a low socio-economic background, diverse cultural group with no insurance or government-sponsored plans like Medicare and Medicaid [13-17]. Patients who come to the resident clinic have poor compliance, increased no-show rates (5%-55%), and are one of the highest risk populations [18-21].

The RITE-BP trial showed that patients who enrolled in the study, irrespective of which group they belonged to had significantly better blood pressures compared to the control group at the end of six months. The patients in the control group belonged to the same population group as the study patients with similar baseline characteristics.

Our study also showed that there was no statistical difference in blood pressures among the patients who had changes made to their medications over the phone and those who hadn’t. What is even more surprising was that in the medication group only one patient’s medication was changed over the phone. Over the six-month period of the study no medication changes were made by the regular primary care providers for the patients enrolled in the study. The common denominator for all the groups in our study population was the device given to measure their blood pressures and counseling on exercise and diet.

We believe that providing a patient with blood pressure monitoring devices, intense coaching on diet and exercise given at the initial visit, and knowledge that a provider was closely following their blood pressures as a psychologically motivating factor were responsible for better blood pressure values in our study subjects compared to the control group. Positive affirmation and psychology have been previously shown to be strong motivating factors to help achieve better blood pressure control [22-24].

Our study has several limitations. As medication changes were being made to the study, participants and investigators were not blinded. The study was done and data were analyzed before the results of the SPRINT trial were published, although currently the JNC VIII guidelines are still in vogue on which our study is based. Of the initial 183 patients that we approached, only 58 agreed to join our trial. One may argue that the patients who volunteered for our study were motivated enough to ensure medication, dietary, and exercise compliance which resulted in the results that we were able to achieve. Our study also had a very low number completing the trial. This is in line with generally patients having poor follow up rates at resident-run clinics and issues with compliance. The no-show rate at our clinic is around 25%-35%. Our sample size was small. We also relied on our patients to provide accurate manual blood pressure readings, which we believe our patients did provide honestly; however, there is always a chance that they could be just giving us random numbers. But we followed up with the patients at three and six months and their logs and blood pressure readings in the clinic reflected the change just as they had said. Our follow up period was short and it would be interesting to follow these patients to see if there has been any residual effect due to our trial.

There are several future directions that the trial can take to further analyze this issue. The use of mobile phone apps has increased in recent years and the use of an algorithm to remind patients to check their blood pressures and take their medications could help. One would also like to analyze the economic impact of utilizing residents and the overall manpower required to help achieve better blood pressure control.

## Conclusions

In conclusion, our trial was able to show that residents were able to achieve a significant drop in systolic and diastolic blood pressures of the patients who were motivated, received regular phone calls, had a blood pressure measuring device, and received intense counseling on exercise and dietary habits compared to the usual standard of care groups. Perhaps following these steps could help us curb this ever-growing problem.
